# Imaging of atherosclerosis, targeting LFA-1 on inflammatory cells with ^111^In-DANBIRT

**DOI:** 10.1007/s12350-018-1244-5

**Published:** 2018-03-13

**Authors:** E. J. Meester, B. J. Krenning, R. H. de Blois, J. P. Norenberg, M. de Jong, M. R. Bernsen, K. Van der Heiden

**Affiliations:** 1 Department of Biomedical Engineering, Thorax Center, Erasmus Medical Center, PO Box 2040, 3000 CA Rotterdam, The Netherlands; 2 Department of Radiology & Nuclear Medicine, Erasmus Medical Center, Rotterdam, The Netherlands; 3 Department of Cardiology, Thorax Center, Erasmus Medical Center, Rotterdam, The Netherlands; 4 Radiopharmaceutical Sciences, University of New Mexico, Albuquerque, NM USA

**Keywords:** Atherosclerosis, inflammation, SPECT, molecular imaging

## Abstract

**Background:**

^111^In-DOTA-butylamino-NorBIRT (DANBIRT) is a novel radioligand which binds to Leukocyte Function-associated Antigen-1 (LFA-1), expressed on inflammatory cells. This study evaluated ^111^In-DANBIRT for the visualization of atherosclerotic plaque inflammation in mice.

**Methods and Results:**

ApoE^−/−^ mice, fed an atherogenic diet up to 20 weeks (n = 10), were imaged by SPECT/CT 3 hours post injection of ^111^In-DANBIRT (~ 200 pmol, ~ 40 MBq). Focal spots of ^111^In-DANBIRT were visible in the aortic arch of all animals, with an average Target-to-Background Ratio (TBR) of 1.7 ± 0.5. In vivo imaging results were validated by ex vivo SPECT/CT imaging, with a TBR up to 11.5 (range 2.6 to 11.5). Plaques, identified by Oil Red O lipid-staining on excised arteries, co-localized with ^111^In-DANBIRT uptake as determined by ex vivo autoradiography. Subsequent histological processing and in vitro autoradiography confirmed ^111^In-DANBIRT uptake at plaque areas containing CD68 expressing macrophages and LFA-1 expressing inflammatory cells. Ex vivo incubation of a human carotid endarterectomy specimen with ^111^In-DANBIRT (~ 950 nmol, ~ 190 MBq) for 2 hours showed heterogeneous plaque uptake on SPECT/CT, after which immunohistochemical analysis demonstrated co-localization of ^111^In-DANBIRT uptake and CD68 and LFA-1 expressing cells.

**Conclusions:**

Our results indicate the potential of radiolabeled DANBIRT as a relevant imaging radioligand for non-invasive evaluation of atherosclerotic inflammation.

**Electronic supplementary material:**

The online version of this article (10.1007/s12350-018-1244-5) contains supplementary material, which is available to authorized users.

## Introduction

Cardiovascular disease remains the major cause of death worldwide.^1^ Most cardiovascular events are a consequence of atherosclerosis, in which plaques form due to chronic inflammation and lipid accumulation in the vessel wall. Plaque progression can result in plaque rupture and subsequent thrombus formation, potentially leading to myocardial infarction or stroke. Timely detection of plaque, before rupture, would allow targeted treatment and prevention of a potentially life-threatening cardiovascular event.

Inflammation is a major hallmark of atherosclerosis and a consistent predictor of cardiovascular risk.^2^,^3^ The recent Canakinumab Anti-inflammatory Thrombosis Outcomes Study (CANTOS) trial showed that reduction of inflammation in patients after myocardial infarction could reduce the risk of new adverse cardiovascular events in the future.^4^ Assessment of the severity of inflammation in atherosclerosis using biomarkers is cumbersome however. High-sensitive C-reactive protein (hsCRP) is a prototypic marker of inflammation and a strong independent predictor for future cardiovascular events, yet is influenced by risk factors such as hypertension, smoking, and periodontal disease.^5^ Non-invasive visualization of arterial inflammation may complement biomarkers such as hsCRP in guiding treatment with new anti-inflammatory drugs.

2-deoxy-2-^18^F-fluoro-d-glucose positron emission tomography (^18^F-FDG PET) imaging is an established method to non-invasively detect and quantify inflammation within atherosclerotic plaques.^6^–^8^ FDG is a glucose analogue, which accumulates in metabolically active cells, including plaque-associated macrophages. However, due to the high metabolic activity of myocardial cells, ^18^F-FDG uptake by the myocardium might hinder detection of coronary atherosclerotic plaques. Furthermore, a necessary fasting period before imaging and non-specific uptake limit the applicability of ^18^F-FDG.^9^,^10^ These limitations warrant the search for additional radioligands which might complement ^18^F-FDG.

**Figure 1:**
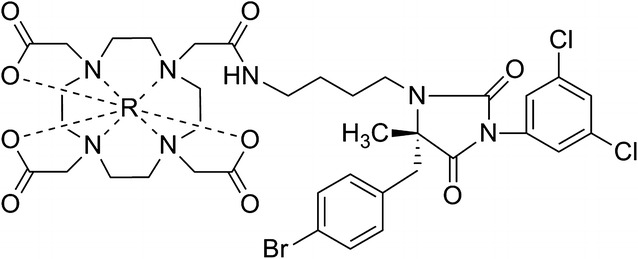
Chemical structure of *R-DANBIRT. 1-(4-aminobutyl)-5-(4-bromobenzyl)-3-(3,5-dichlorophenyl)-5-methylimidazolidine-2,4-dione-DOTA. *R*,radiometal

Development of atherosclerosis is initiated by adhesion of monocytes as well as T lymphocytes to the arterial endothelial surface, followed by their migration into the subendothelial space.^11^ Leukocyte Function-associated Antigen-1 (LFA-1), consisting of CD11a and CD18 subunits, is an integrin cell-surface receptor expressed on leukocytes. LFA-1 binds to endothelial cells via interaction with Intercellular Adhesion Molecule 1 (ICAM-1), and is involved in transmigration of inflammatory cells to sites of inflammation.^12^ LFA-1 was first identified as one of the several adhesion molecules playing a role in leukocyte trafficking, antigen presentation, and cell activation. Immunohistochemical studies on animal models of atherosclerosis have demonstrated the presence of LFA-1-positive cells in atherosclerotic lesions.^13^,^14^ Therefore, LFA-1 may be a promising imaging target for atherosclerotic plaque detection. Recently, an allosteric inhibitor of LFA-1^15^ was chemically adapted for nuclear diagnostic and therapeutic purposes ((R)-1-(4-aminobutyl)-5-(4-bromobenzyl)-3-(3,5-dichlorophenyl)-5-methylimidazolidine-2,4-dione; butylamino-NorBIRT).^16^ Butylamino-NorBIRT can be labeled with various radionuclides for Single Photon Emission Computed Tomography (SPECT) and PET imaging via a chelator (e.g., 1,4,7,10-tetraazacyclododecane-1,4,7,10-tetraacetic acid (DOTA)), depicted in Figure 1. Indium-111-DOTA-butylamino-NorBIRT (^111^In-DANBIRT) is used for SPECT imaging and has been shown to specifically bind LFA-1 on cultured human leukemic and mouse lymphoma cells.^16^,^17^

The aim of this study was to examine the feasibility of plaque detection using ^111^In-DANBIRT. To this end, we studied in vivo uptake in ApoE^−/−^ mice via SPECT/Computed Tomography (CT) scanning, and established its binding to human plaque material.

## Materials and Methods

### Animals and Experimental Setup

Female ApoE ^−/−^ mice on a C57BL/6 J background (*n* = 10) were purchased from Charles Rivers (Calco, Italy) at 6 weeks of age and were fed a high fat diet (0.3% cholesterol, Altromin Spezialfutter GmbH & Co. KG, Lage, Germany) ad libitum from 8 weeks of age onwards up to a maximum of 20 weeks. After SPECT/CT imaging, mice were sacrificed by an overdose of inhalation anesthesia (isoflurane). All animal experiments were approved by the institutional animal studies committee and were in accordance with Dutch animal ethical legislation and the European Union Directive.

### Radiolabeling

^111^In-DANBIRT (MW = 886.5 g/mole) (provided by J.P. Norenberg) was radiolabeled with ^111^In (Covidien, Petten, The Netherlands) with a specific activity of 200 MBq/nmol, as previously described.^18^ Radiochemical purity (> 90%) and incorporation yield (> 99%) were assessed using high-pressure liquid chromatography and instant thin-layer chromatography on silica gel. Quenchers (3.5 mM ascorbic acid, 3.5 mM gentisic acid, 10 mM methionine) were added to prevent radiolysis of the tracer.^19^

### In vivo Imaging

Mice were injected intravenously in the tail vein with ~ 40 MBq/200 pmol ^111^In-DANBIRT in 0.1% Bovine serum albumin (BSA) in phosphate-buffered saline (PBS) (injection volume of 150 µL) (*n* = 10). They were anesthetized (1.5 to 2% isoflurane) 2.5 h post injection, after which they were injected with 100 µL CT-contrast agent (eXIA160, Binito Biomedical Inc, Ottawa, ON, Canada; or Exitron nano 12000, Milteny Biotec, Bergisch-Gladach, Germany). Immediately after contrast injection, a CT scan was performed (17 min, 615 mA, 55 keV) followed by a SPECT scan (static scan, 1 h, 2.0-mm pinhole collimator, 17 positions) on a hybrid SPECT/CT scanner (VECTor, MILabs, Utrecht, The Netherlands) with a reported spatial resolution of 0.85 mm.^20^ SPECT images were reconstructed using photopeak windows of 214-262 and 152-185 keV, with a background window on either side of the photopeak with a width of 2.5% of the corresponding photopeak, and a pixel-based ordered subset expectation maximization method (4 subsets and 30 iterations), a voxel size of 0.8 mm^3^, and corrected for attenuation using the CT data. A post-reconstruction 3-dimensional Gaussian filter was applied (0.8 mm full width at half maximum). A blocking study was performed by co-injection with a 100× or 850× excess of unlabeled DANBIRT in two mice.

### Ex vivo Studies

After imaging, mice were euthanized and vasculature was flushed with PBS via the left ventricle, followed by excision of the aorta and carotid arteries. The arteries were cleaned from visceral fat and connective tissue and stained for lipids (Oil red O (ORO) according to standard protocol) to confirm plaque presence. After staining, the arteries were scanned ex vivo (CT: 5 min, 615 mA, 55 keV; SPECT: static scan, 30 min, 2.0-mm pinhole collimator) on a SPECT/CT scanner (VECTor, MILabs, Utrecht, The Netherlands). SPECT images were reconstructed as described above. Subsequently, arteries were used for ex vivo autoradiography (*n* = 4) or embedded in tissue-tec and stored at – 80 °C for histological analysis (*n* = 6). To determine the biodistribution of ^111^In-DANBIRT in ApoE^−/−^ mice, selected organs were collected, weighed, and measured for radioactivity in a gamma-counter (1480 Wizard, Gamma counter, Perkin Elmer). Radioactivity measured in the various tissues was expressed as percentage of injected dose per gram (%ID/g). ^111^In-DANBIRT showed a biodistribution pattern according to expectation, with limited retention in major organs 24 hours post injection (Online Resource 1). After ~ 3 weeks, arteries used for ex vivo autoradiography were placed on a phosphor screen overnight and read using a phosphor imager (Cyclone; Perkin Elmer). Plaque signal was quantified as digital light units/mm^2^ (DLU/mm^2^) using Optiquant software (Perkin Elmer), and compared to non-diseased arterial signal in the same sample to calculate a Target-to-Background Ratio (TBR).

### Ex vivo Carotid Endarterectomy Study

To explore binding to human atherosclerotic plaques, we incubated a human carotid endarterectomy (CEA, acquired with informed consent and approved by the medical ethics committee) sample with ^111^In-DANBIRT (~ 950 pmol, ~ 190 MBq) in 0.1% BSA in PBS for 2 hours. After incubation, the specimen was washed in PBS and subsequently scanned on the SPECT/CT scanner (CT: 5 min, 615 mA, 55 keV; SPECT: static scan, 30 min, 2.0-mm pinhole collimator). SPECT images were reconstructed as described above. Hereafter, the specimen was cut in transverse slices of 1 mm. Even slices were used for autoradiography, odd slice for histological evaluation. Histology was visually matched with transverse views of the SPECT/CT scan, to correlate ^111^In-DANBIRT uptake with locations of cells expressing LFA-1 and CD68.

### Immunohistochemistry and In vitro Autoradiography

Mouse aortic arch cryosections and human CEA cryosections (both 5 µm) were immunohistochemically stained with anti-CD68, a pan-macrophage marker (human: 1:100, Abcam, ab955; mouse: 1:100 Biorad, MCA1957), and anti-LFA-1 (human: 1:100 Biorad, MCA1848; mouse: 1:1, Pont et al. 1986^21^) to assess co-localization of LFA-1 and CD68 (macrophage)-positive cells. In vitro autoradiography was performed on mouse cryosections (10 µm) of aortic arch adjacent to slides used for immunohistochemistry, by incubating slides for 1 hour with 80 µL 10^−9^ M ^111^In-DANBIRT. Serially sectioned human CEA cryosections (10 µm) were incubated for 1 h with 80 µL 10^−9^ M ^111^In-DANBIRT with or without excess (10^−6^ M) unlabeled DANBIRT to determine non-specific binding. Slides were exposed to phosphor screens for 1 week and read using a phosphor imager (Cyclone; Perkin Elmer). The autoradiograms of human CEA cryosections were analyzed by drawing fixed regions of interests (ROIs) around the sections using Optiquant software (Perkin Elmer). Signal was quantified as DLU/mm^2^, after which blocked and unblocked sections were compared using a student’s *t* test.

### Quantification

SPECT data were analyzed with Vivoquant (Invicro) by quantification of manually drawn regions of interests (ROIs) based on contrast-enhanced CT. On in vivo scans, ROIs were drawn in the aortic arch, vena cava inferior, and jugular vein. ROIs on ex vivo scans were drawn in the aortic arch and non-diseased artery in the descending aorta or common carotid arteries. TBRs were calculated, and expressed as mean ± standard deviation.

## Results

### In vivo Mouse Plaque Imaging

**Figure 2:**
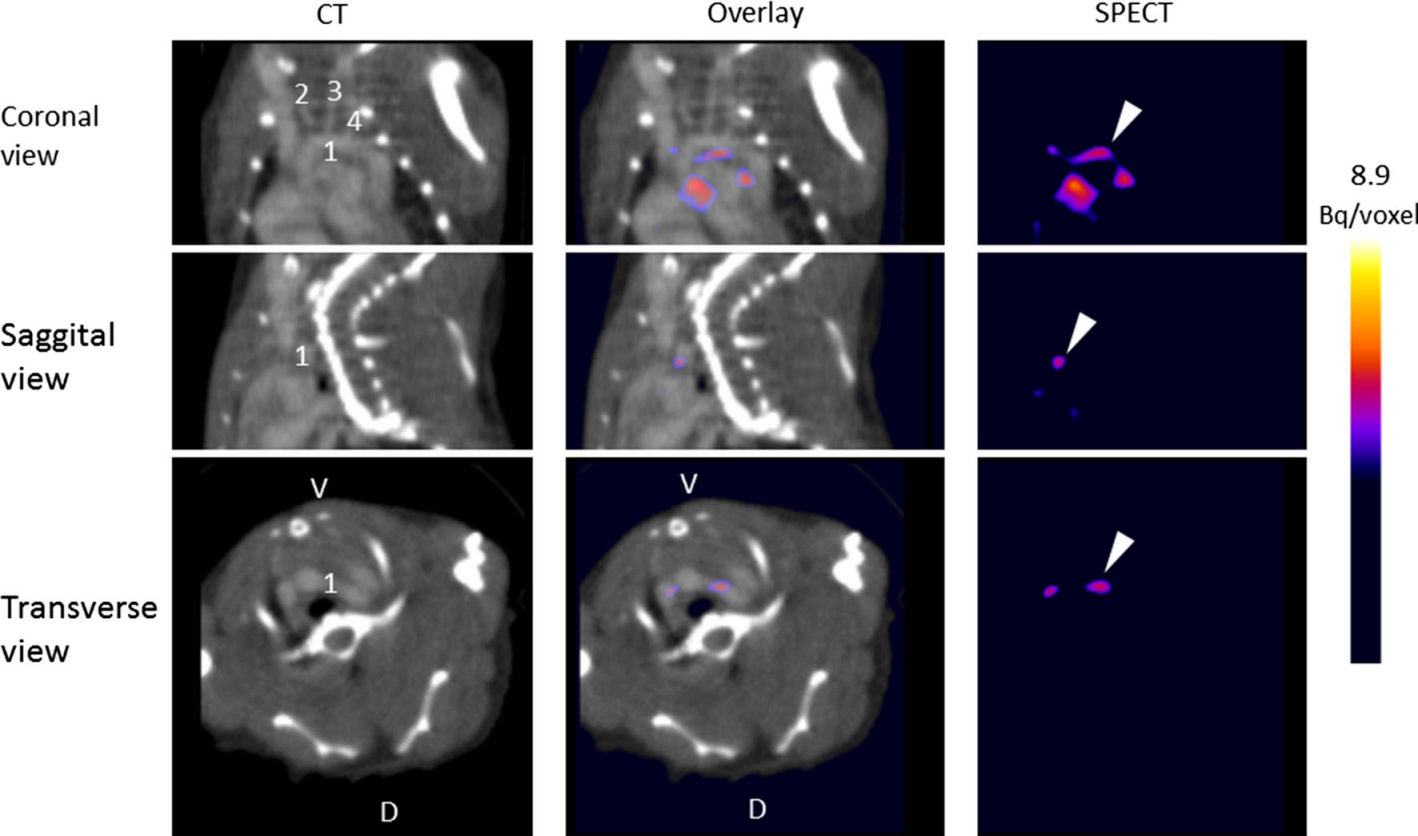
In vivo uptake of ^111^In-DANBIRT in atherosclerotic mice. In vivo contrast-enhanced Computed Tomography (CT), Single Photon Emission Computed Tomography (SPECT), and overlay images of ApoE^−/−^ mouse thorax in coronal, sagittal, and transverse view. Scans were made 3 h post injection. Exitron nano 12000 was used as contrast agent. *1* aortic arch; *2* right common carotid artery; *3* left common carotid artery; *4* left subclavian artery. *V*, ventral; *D*, dorsal. Arrowheads indicate radioactive hot spots (^111^In-DANBIRT uptake) in the aortic arch

In vivo SPECT/CT imaging 3 hours after intravenous injection of ^111^In-DANBIRT showed distinct focal spots of radioactivity, corresponding to common sites of plaque formation in the aortic arch of all animals (Figure 2 and Online Resource 4), with an average TBR of 1.7 ± 0.5. Uptake of ^111^In-DANBIRT at plaque locations in the carotid bifurcations was not above background. Myocardial uptake was not above background in any of the animals. Co-injection of excess unlabeled DANBIRT reduced uptake of ^111^In-DANBIRT in plaque areas of the aortic arch to background levels (Online Resource 2).

### Ex vivo Validation

**Figure 3:**
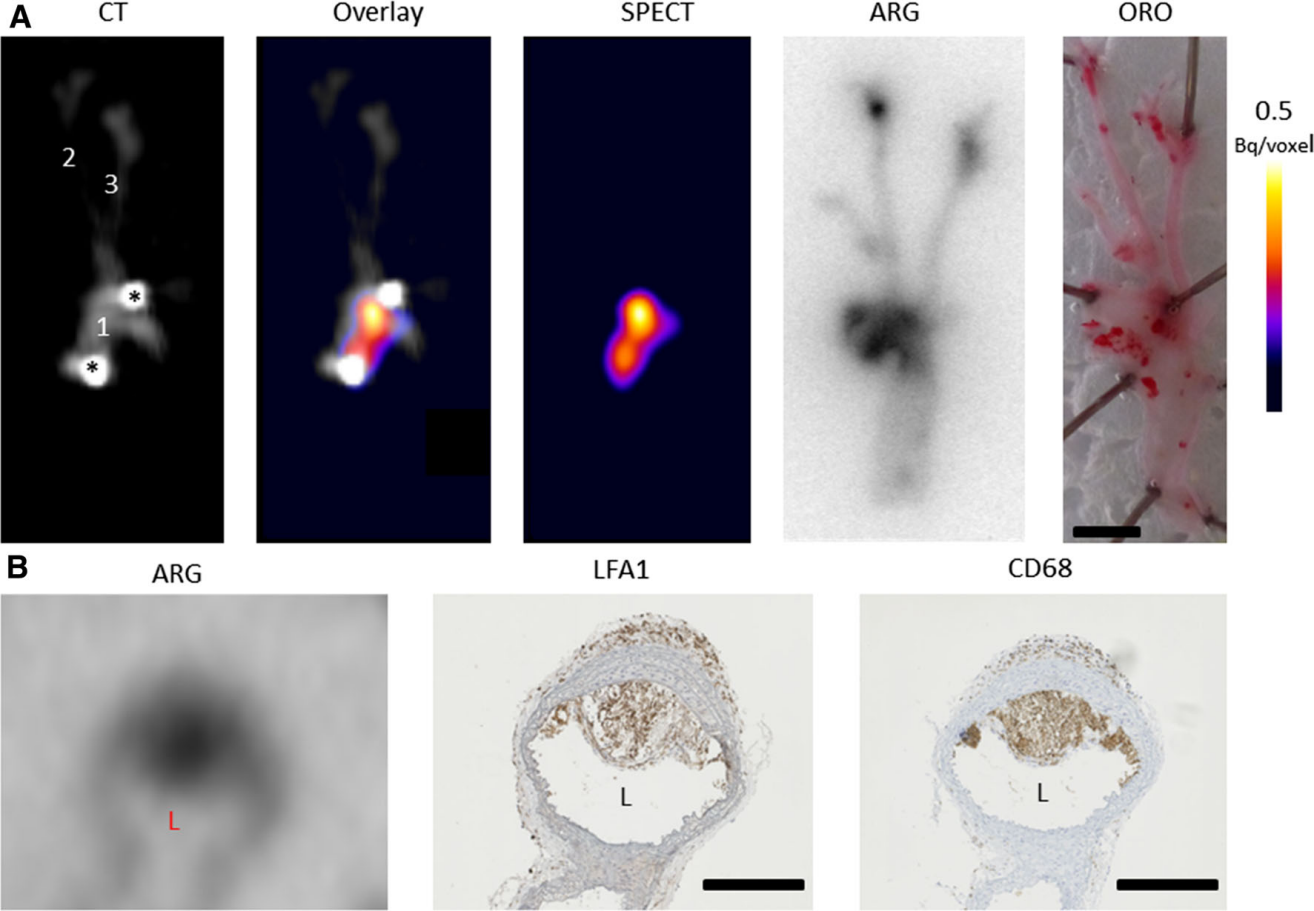
Ex vivo validation of ^111^In-DANBIRT imaging of LFA-1. Frontal view of ex vivo CT, SPECT, and overlay images of an excised artery of an ApoE^−/−^ mouse, autoradiography (ARG) and bright field photo of Oil Red O (ORO) stained, opened artery excised from another ApoE^−/−^ mouse, both injected with ^111^In-DANBIRT. *1* aortic arch; *2* right common carotid artery; *3* left common carotid artery. Asterisks (*) indicate locations of needles used to pin down the artery. Scale bar = 2 mm (panel **A**). In vitro autoradiography of 10-µm cryosection of mouse aortic arch. Adjacent 5-µm cryosections immunohistochemically stained for LFA-1 and CD68 expression. Scale bar = 100 µm (panel **B**), L = Lumen

After in vivo imaging, arteries were excised and scanned ex vivo. Focal uptake of ^111^In-DANBIRT in the aortic plaques was evident (Figure 3A), with a TBR up to 11.5 (range 2.6-11.5). To co-localize uptake to plaque locations, we performed ex vivo autoradiography and a lipid staining (ORO) on the excised arteries. The ex vivo autoradiography showed high ^111^In-DANBIRT uptake in regions in the aortic arch, arterial branch points, descending aorta, and carotid bifurcations, corresponding to plaque locations as indicated by lipid staining (Figure 3A) with an average TBR of 19.0 ± 7.9. In vitro autoradiography and immunohistochemistry of mouse aorta containing plaque demonstrated ^111^In-DANBIRT binding to plaque areas containing LFA-1-expressing inflammatory cells and CD68-positive macrophages (Figure 3B). Binding of ^111^In-DANBIRT to human plaque cryosections could be blocked by addition of an excess of unlabeled DANBIRT (Online Resource 3).

### Human Plaque Imaging

**Figure 4:**
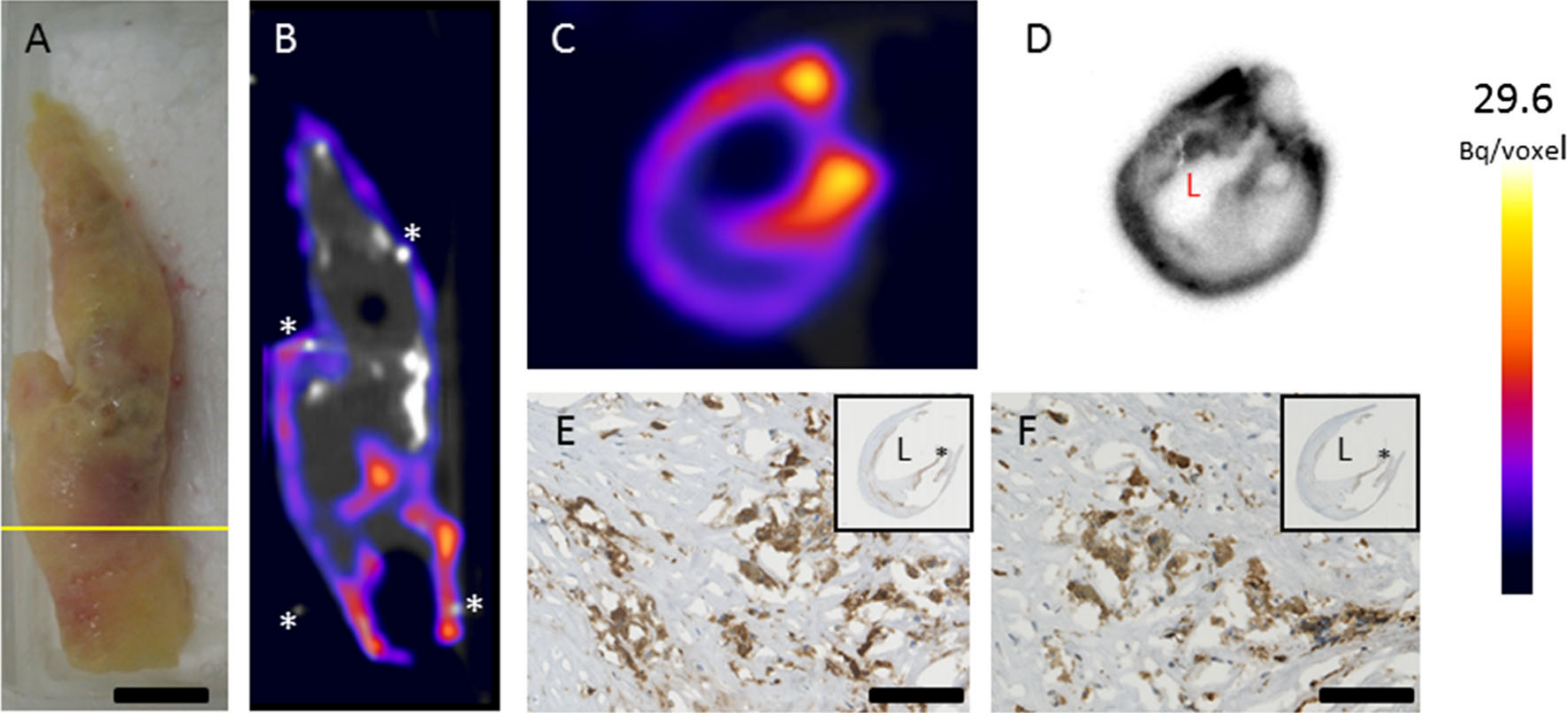
Ex vivo ^111^In-DANBIRT imaging of LFA-1 in a human atherosclerotic plaque. Bright field photo of a Carotid Endarterectomy specimen. Yellow line indicates location of transverse view of SPECT/CT scan in C, autoradiography of 1 mm thick transverse slide in D, and adjacent histologic sections in E and F. Scale bar = 10 mm (**A**). SPECT/CT imaging shows heterogeneous uptake of ^111^In-DANBIRT. Calcified regions of the plaque are visible in bright white in the CT views. Pins used as landmarks are indicated by asterisks (*). Coronal (**B**), and transverse views (**C**) of SPECT/CT scan. Ex vivo autoradiography image (**D**). LFA-1 expression is visible in a slide adjacent to D, co-localizing with areas of high ^111^In-DANBIRT uptake. Inset shows overview of the histological section; asterisk shows location of zoomed area. Scale bar = 100 µm (**E**). CD68 expressing macrophages are present in an adjacent cryosection. Inset shows overview of the histological section; asterisk shows location of zoomed area. Scale bar = 100 µm (**F**), L = Lumen

Incubation of a human CEA sample with ^111^In-DANBIRT resulted in heterogeneous uptake of ^111^In-DANBIRT seen as hot spots throughout the plaque in SPECT/CT scans (Figure 4A, B, and Online Resource 5). Subsequent histological analysis of adjacent cryosections demonstrated co-localization of the hotspots of ^111^In-DANBIRT uptake with LFA-1-expressing inflammatory cells and CD68-positive macrophages (Figure 4C–F).

## Discussion

In this study, we show the feasibility of in vivo plaque detection using ^111^In-DANBIRT SPECT/CT imaging of LFA-1. We demonstrated distinct focal uptake in the aortic arch of all animals 3 hours after intravenous injection of ^111^In-DANBIRT, despite the small dimensions of murine arteries and plaques. This uptake was related to the presence of lipid rich plaques with inflammatory cells shown by ORO staining and immunohistochemistry. Moreover, we showed uptake of ^111^In-DANBIRT in an excised human carotid plaque. In addition, the presence of LFA-1-positive cells in atherosclerotic plaque was confirmed by immunohistochemistry. The finding that ^111^In-DANBIRT can detect plaques in murine aortas (vessel diameter < 1 mm) point towards its possible applicability in human coronary arteries which have a common diameter of around 3 mm^22^.

Various non-invasive imaging techniques are explored for the early detection of atherosclerosis and risk stratification of patients with cardiovascular disease, using various target-ligand combinations that are associated with atherosclerosis. The clinically most frequently explored technique is ^18^F-FDG PET, which has limits in regard to its specificity and myocardial uptake. DANBIRT, in this study labeled with ^111^In, solely binds to inflammatory cells and was not taken up by the myocardium in our atherosclerotic mouse model. As such, DANBIRT might surpass a limitation of ^18^F-FDG. Moreover, DANBIRT might provide information on different stages of plaque development as all leukocytes express LFA-1. Furthermore, DANBIRT can be labeled with ^68^Ga, which makes it suitable for PET imaging and enhances its clinical applicability.

^111^In-DANBIRT signal was not visible by in vivo nor ex vivo SPECT/CT imaging of the plaque in the carotid artery bifurcations, whereas the more sensitive ex vivo autoradiography did demonstrate uptake. This could be due to a smaller plaque size and/or a lower number, or a more diffuse distribution of inflammatory cells, resulting in a signal below the detection threshold of our SPECT system.

^111^In-DANBIRT exhibits specific uptake in plaques in atherosclerotic mice. Uptake of ^111^In-DANBIRT occurs in plaque areas that contain LFA-1 expressing leukocytes and CD68-positive macrophages, indicating specific targeting of LFA-1-positive cells. We conclude that radiolabeled DANBIRT shows potential as a relevant nuclear imaging ligand to detect atherosclerotic inflammation. Upon confirmation of these findings in a patient population, this imaging tool may be used to improve risk assessment of cardiovascular disease by visualizing inflammation and for individualizing specific anti-inflammatory drug therapy.

## New Knowledge Gained

The novel LFA-1 targeting radioligand DANBIRT detects inflammation in vivo in atherosclerotic plaques in an ApoE^−/−^ mouse model. DANBIRT uptake correlates to the presence of CD68 expressing macrophages and LFA-1 expressing inflammatory cells in the atherosclerotic plaque. In addition, ex vivo uptake of DANBIRT in a human carotid plaque correlates to the presence of CD68 expressing macrophages and LFA-1 expressing inflammatory cells, suggesting the potential of DANBIRT for non-invasive imaging of atherosclerotic plaque inflammation.

## Electronic supplementary material

Below is the link to the electronic supplementary material.

Supplementary material 1 (DOCX 306 kb)

Supplementary material 2 (GIF 2089 kb)

Supplementary material 3 (GIF 3566 kb)

Supplementary material 4 (PPTX 862 kb)

## Abbreviations

LFA-1: Leukocyte function-associated antigen-1
SPECT: Single photon emission computed tomography
CT: Computed tomography
PET: Positron emission tomography
^18^F-FDG: 2-deoxy-2-^18^F-fluoro-d-glucose
DANBIRT: ^111^In-DOTA-butylamino-NorBIRT
ApoE: Apolipoprotein E
CEA: Carotid endarterectomy
ROI: Region of interest
TBR: Target-to-background ratio
