# Supplementary material for: Imaging of atherosclerosis, targeting LFA-1 on inflammatory cells with 111In-DANBIRT
Source: J Nucl Cardiol. 2018 Mar 13;26(5):1697–704. doi: 10.1007/s12350-018-1244-5 (PMC6775031; doi:10.1007/s12350-018-1244-5)
Supplement: Supplementary file 1 — Supplementary material 1 (DOCX 306 kb) [file 12350_2018_1244_MOESM1_ESM.docx]

**Supplemental Material**

**Imaging of atherosclerosis, targeting LFA-1 on inflammatory cells with ^111^In-DANBIRT**

*Journal of Nuclear Cardiology*

E.J. Meester^1,2^ Msc; B.J. Krenning^3^ MD, PhD; R.H. de Blois^2^ PhD; J.P. Norenberg^4^ PhD, PharmD; M. de Jong^2^ PhD; M.R. Bernsen^2^ PhD; K van der Heiden^1*^ PhD

^1^ Department of Biomedical Engineering, Thorax Center, Erasmus MC, Rotterdam, The Netherlands

^2^ Department of Radiology & Nuclear Medicine, Erasmus MC, Rotterdam, The Netherlands

^3^ Department of Cardiology, Thorax Center, Erasmus MC, Rotterdam, The Netherlands

^4^Radiopharmaceutical Sciences, University of New Mexico, Albuquerque, NM, USA

*Corresponding author

Correspondence address: Dr. Kim van der Heiden, Department of Biomedical Engineering, Erasmus Medical Center, PO Box 2040, 3000 CA Rotterdam, The Netherlands

E-mail: [k.vanderheiden@erasmusmc.nl](mailto:k.vanderheiden@erasmusmc.nl)

Telephone: +31 (0)10 7038166

Fax: +31 (0)10 7044720

**Supplemental Figures**

**
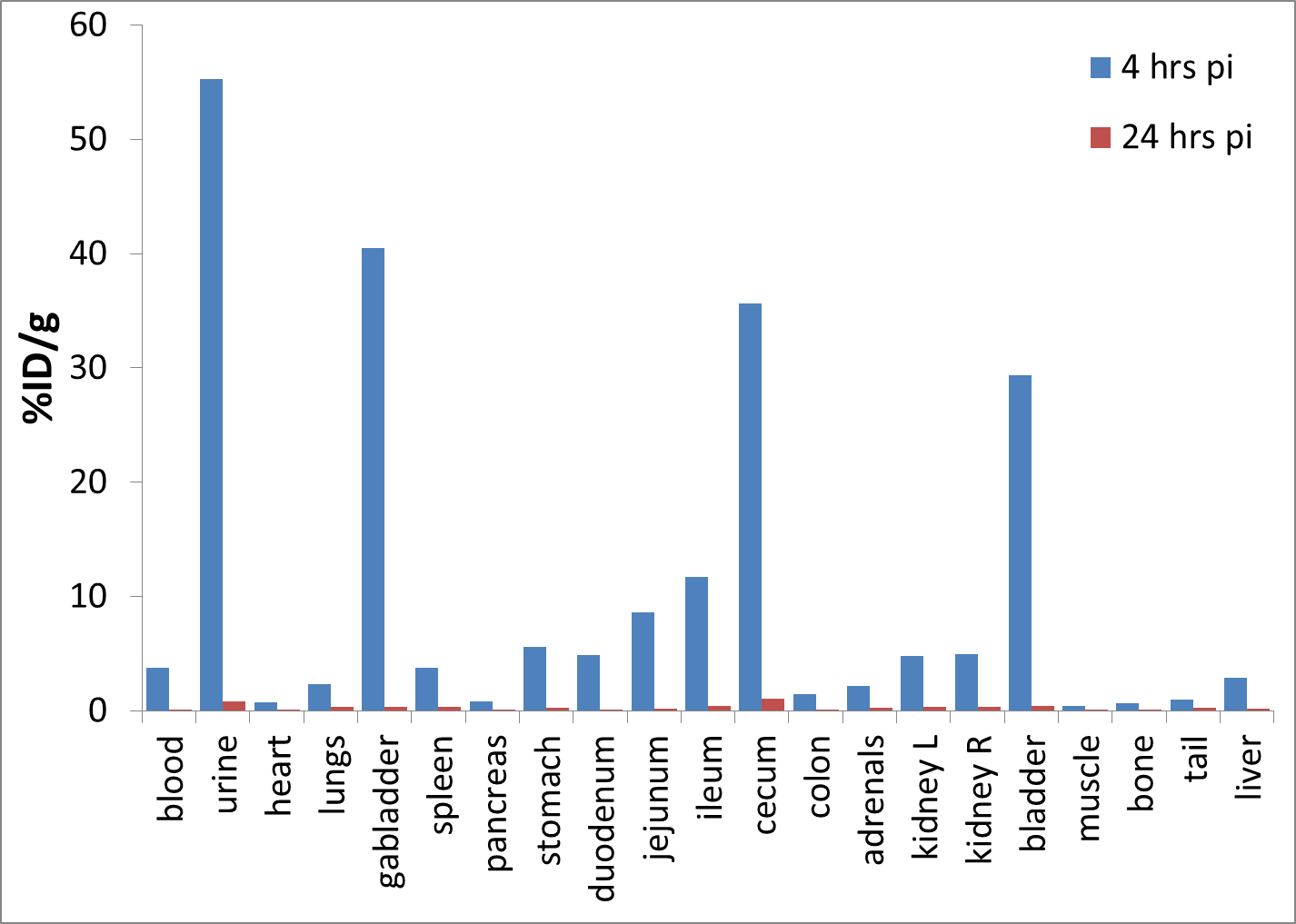
**

Online Resource 1


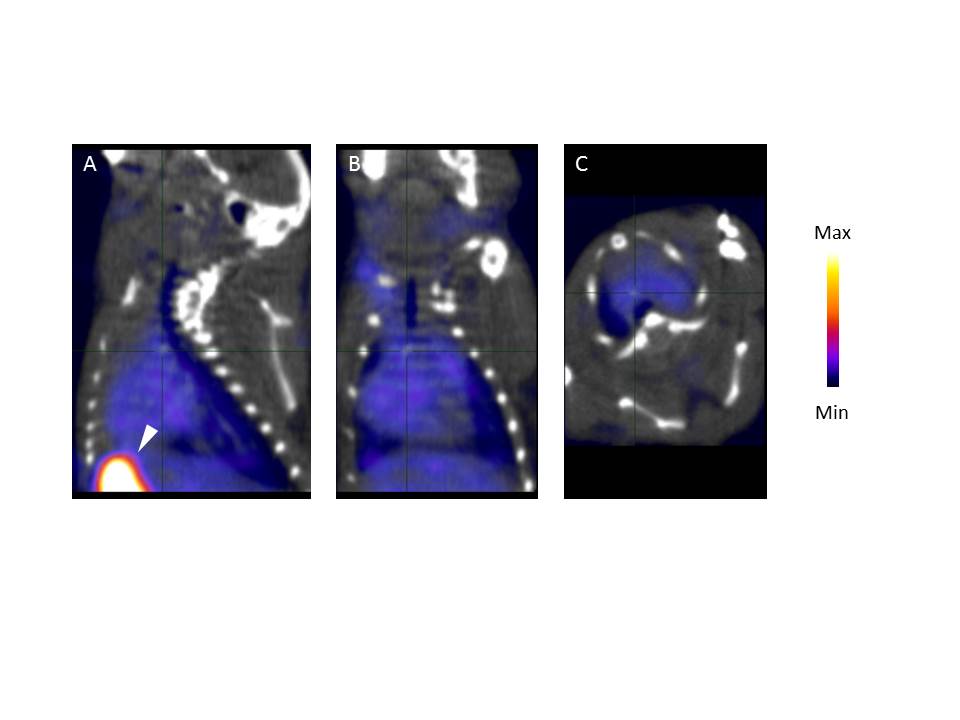


Online Resource 2


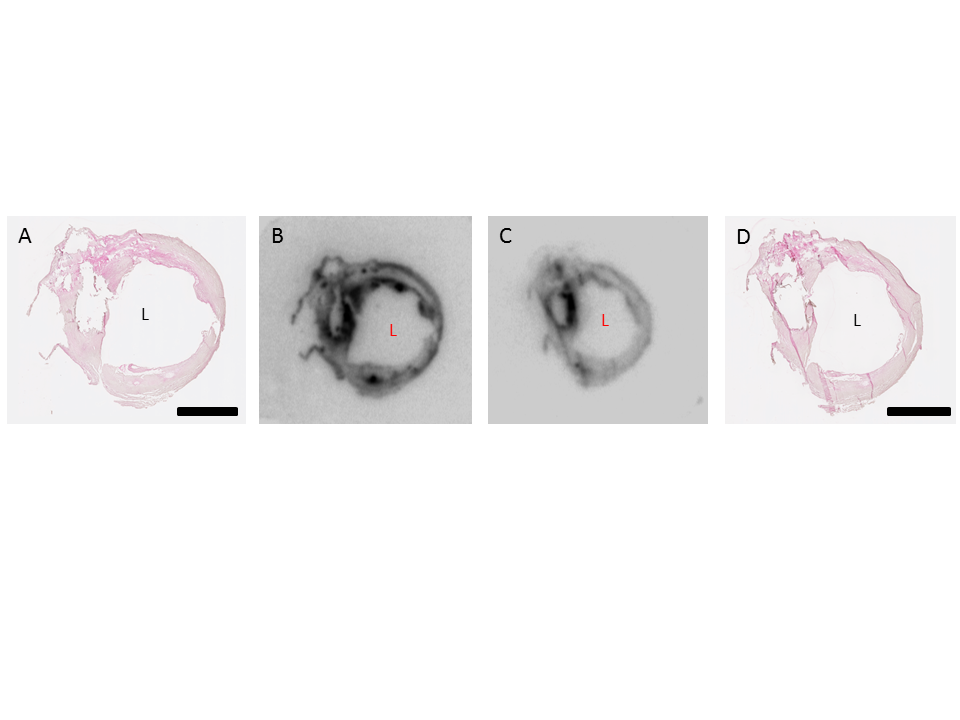
Online Resource 3

**Supplemental Figure and Movie Legends**

**Online Resource 1: Clearance of ^111^In-DANBIRT 24 hours pi.** 4 hours after radioligand injection, high uptake is visible in the gallbladder, liver and intestines, which is suggestive of excretion via the hepatobiliary system. 24 hours pi most ^111^In-DANBIRT has been cleared.

**Online Resource 2: *In vivo* blocking of ^111^In-DANBIRT in atherosclerotic mice.** *In vivo* SPECT/CT images of an ApoE^-/-^ mouse thorax in sagittal (A), coronal (B), and transverse (C) view. Scans were made 3 hrs pi, with an 850x excess of unlabelled DANBIRT co-injected. Calcified plaque area is visible at the crosshair. Plaque presence was confirmed by Oil Red O staining of excised arteries (data not shown). High signal remains visible in the gallbladder (indicated by arrowhead), in line with hepatobiliary excretion. Blocking reduced the target to background ratio to 1.1 and 0.9 (100x and 850x excess unlabelled DANBIRT, respectively).

**Online Resource 3: *In vitro* blocking of ^111^In-DANBIRT in human carotid endarterectomy cryosections.** *In vitro* autoradiography performed with 10^-9^ M ^111^In-DANBIRT on 10µm cryosections of CEA material without (B) or with (C) blocking solution (10^-6^ M unlabelled DANBIRT). A and D show the corresponding sections stained with Haematoxylin-Eosin. DLU/mm^2^ is significantly reduced by blocking (non-blocked=15*10^5^±4.6*10^5^, blocked=3.1*10^5^ ±1.8*10^5^ DLU/mm^2^, p=0.001, n=24). L=Lumen

**Online Resource 4:** 3D movie of focal ^111^In-DANBIRT uptake in the aortic arch of mouse vasculature (grey).

**Online Resource 5:** Movie of *ex vivo* CEA-specimen incubated with ^111^In-DANBIRT, visualized with SPECT/CT. CT shows bright white calcifications, needles used to pin down the sample are visible as white stripes.
