# Supplementary figures and images for: Imaging of atherosclerosis, targeting LFA-1 on inflammatory cells with 111In-DANBIRT
Source: J Nucl Cardiol. 2018 Mar 13;26(5):1697–704. doi: 10.1007/s12350-018-1244-5 (PMC6775031; doi:10.1007/s12350-018-1244-5)

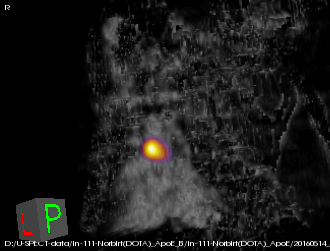

Supplement: Supplementary file 2 — Supplementary material 2 (GIF 2089 kb) [file 12350_2018_1244_MOESM2_ESM.gif]

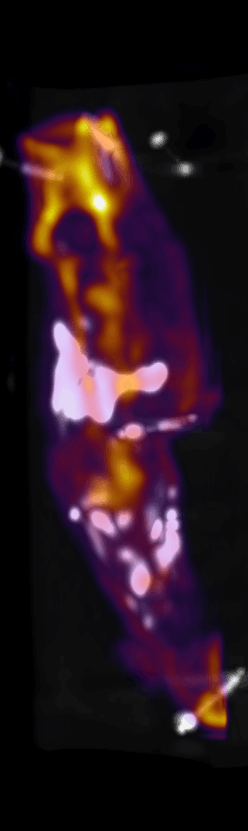

Supplement: Supplementary file 3 — Supplementary material 3 (GIF 3566 kb) [file 12350_2018_1244_MOESM3_ESM.gif]
