# Supplementary material for: Imaging of atherosclerosis, targeting LFA-1 on inflammatory cells with 111In-DANBIRT
Source: J Nucl Cardiol. 2018 Mar 13;26(5):1697–704. doi: 10.1007/s12350-018-1244-5 (PMC6775031; doi:10.1007/s12350-018-1244-5)
Supplement: Supplementary file 4 — Supplementary material 4 (PPTX 862 kb) [file 12350_2018_1244_MOESM4_ESM.pptx]

## Slide 1
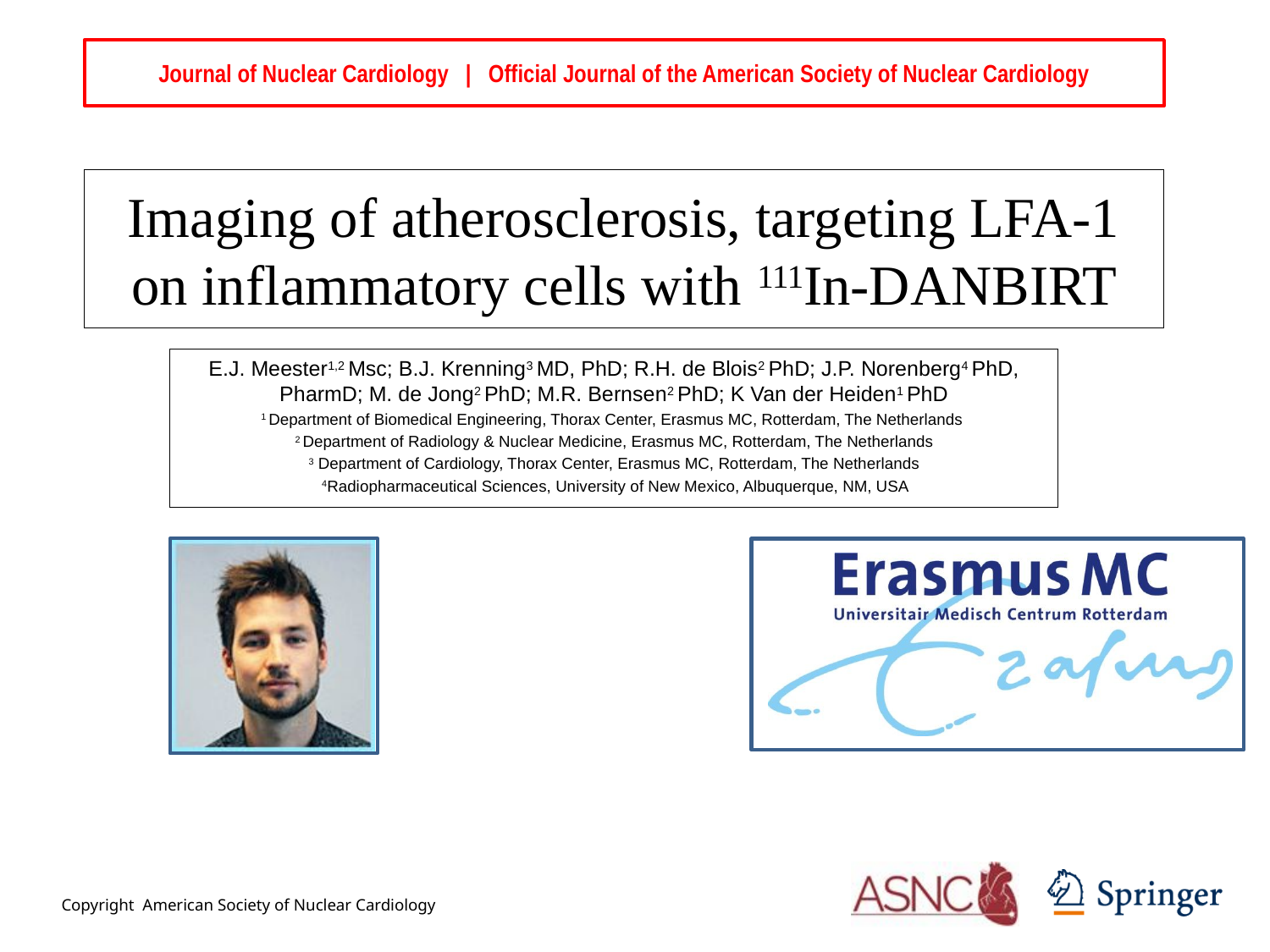

Journal of Nuclear Cardiology | Official Journal of the American Society of Nuclear Cardiology
# Imaging of atherosclerosis, targeting LFA-1 on inflammatory cells with 111In-DANBIRT
E.J. Meester1,2 Msc; B.J. Krenning3 MD, PhD; R.H. de Blois2 PhD; J.P. Norenberg4 PhD, PharmD; M. de Jong2 PhD; M.R. Bernsen2 PhD; K Van der Heiden1 PhD
1 Department of Biomedical Engineering, Thorax Center, Erasmus MC, Rotterdam, The Netherlands
2 Department of Radiology & Nuclear Medicine, Erasmus MC, Rotterdam, The Netherlands
3 Department of Cardiology, Thorax Center, Erasmus MC, Rotterdam, The Netherlands
 4Radiopharmaceutical Sciences, University of New Mexico, Albuquerque, NM, USA
Copyright American Society of Nuclear Cardiology

## Slide 2
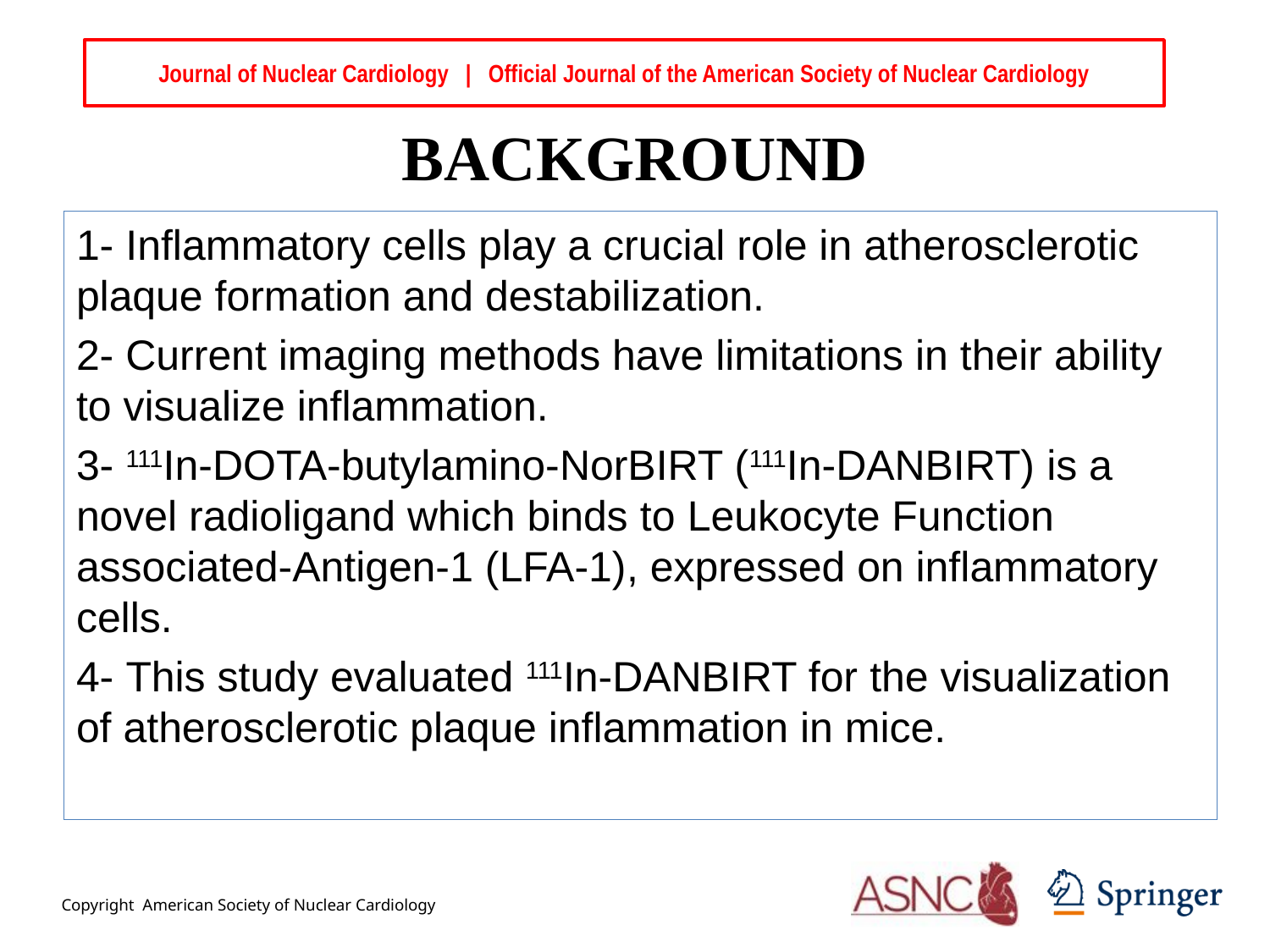

Journal of Nuclear Cardiology | Official Journal of the American Society of Nuclear Cardiology
# BACKGROUND
1- Inflammatory cells play a crucial role in atherosclerotic plaque formation and destabilization.
2- Current imaging methods have limitations in their ability to visualize inflammation.
3- 111In-DOTA-butylamino-NorBIRT (111In-DANBIRT) is a novel radioligand which binds to Leukocyte Function associated-Antigen-1 (LFA-1), expressed on inflammatory cells.
4- This study evaluated 111In-DANBIRT for the visualization of atherosclerotic plaque inflammation in mice.
Copyright American Society of Nuclear Cardiology

## Slide 3
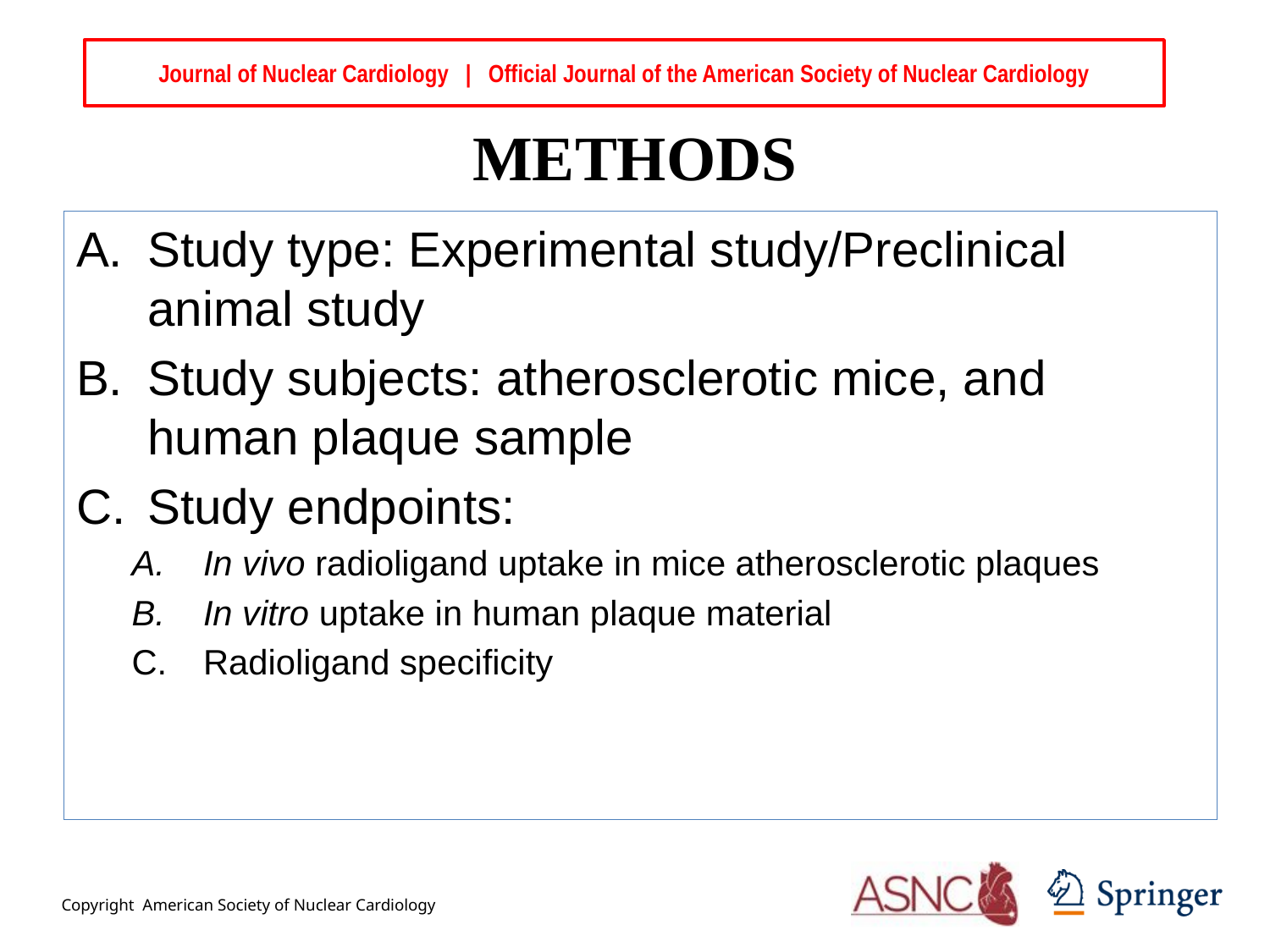

Journal of Nuclear Cardiology | Official Journal of the American Society of Nuclear Cardiology
# METHODS
Study type: Experimental study/Preclinical animal study
Study subjects: atherosclerotic mice, and human plaque sample
Study endpoints:
In vivo radioligand uptake in mice atherosclerotic plaques
In vitro uptake in human plaque material
Radioligand specificity
Copyright American Society of Nuclear Cardiology

## Slide 4
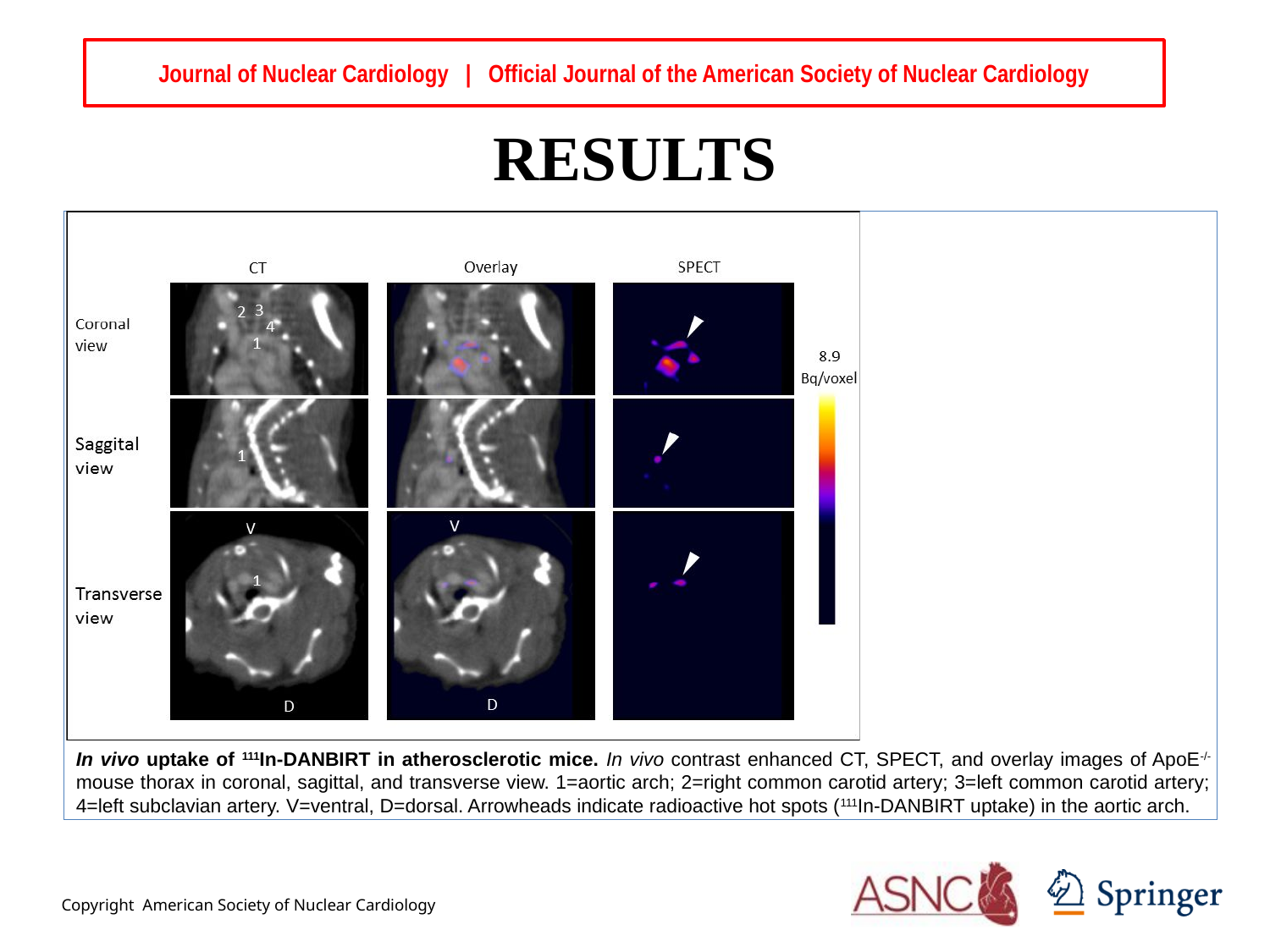

Journal of Nuclear Cardiology | Official Journal of the American Society of Nuclear Cardiology
# RESULTS
In vivo uptake of 111In-DANBIRT in atherosclerotic mice. In vivo contrast enhanced CT, SPECT, and overlay images of ApoE-/- mouse thorax in coronal, sagittal, and transverse view. 1=aortic arch; 2=right common carotid artery; 3=left common carotid artery; 4=left subclavian artery. V=ventral, D=dorsal. Arrowheads indicate radioactive hot spots (111In-DANBIRT uptake) in the aortic arch.
Copyright American Society of Nuclear Cardiology

## Slide 5
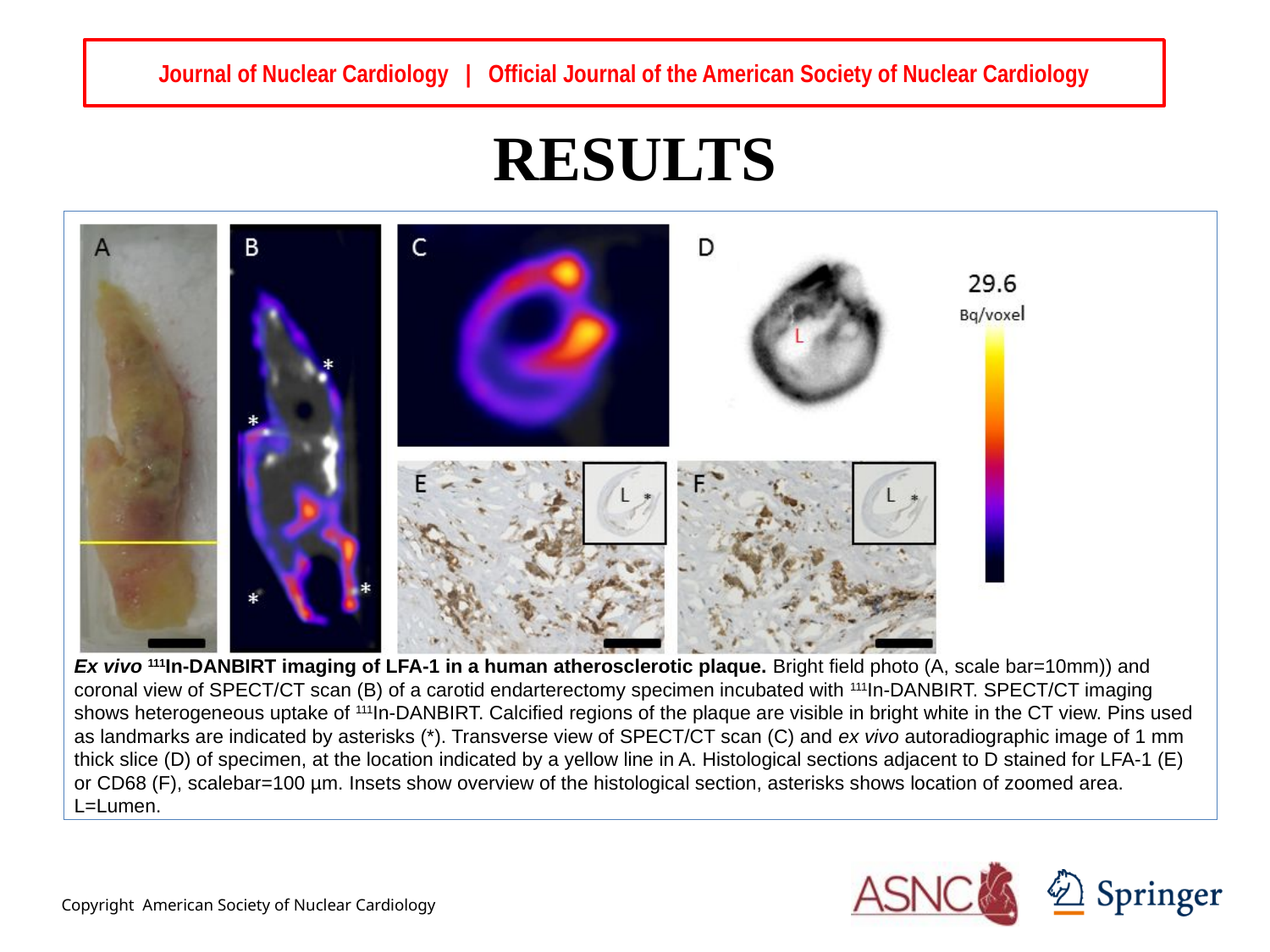

Journal of Nuclear Cardiology | Official Journal of the American Society of Nuclear Cardiology
# RESULTS
Ex vivo 111In-DANBIRT imaging of LFA-1 in a human atherosclerotic plaque. Bright field photo (A, scale bar=10mm)) and coronal view of SPECT/CT scan (B) of a carotid endarterectomy specimen incubated with 111In-DANBIRT. SPECT/CT imaging shows heterogeneous uptake of 111In-DANBIRT. Calcified regions of the plaque are visible in bright white in the CT view. Pins used as landmarks are indicated by asterisks (*). Transverse view of SPECT/CT scan (C) and ex vivo autoradiographic image of 1 mm thick slice (D) of specimen, at the location indicated by a yellow line in A. Histological sections adjacent to D stained for LFA-1 (E) or CD68 (F), scalebar=100 µm. Insets show overview of the histological section, asterisks shows location of zoomed area. L=Lumen.
Copyright American Society of Nuclear Cardiology

## Slide 6
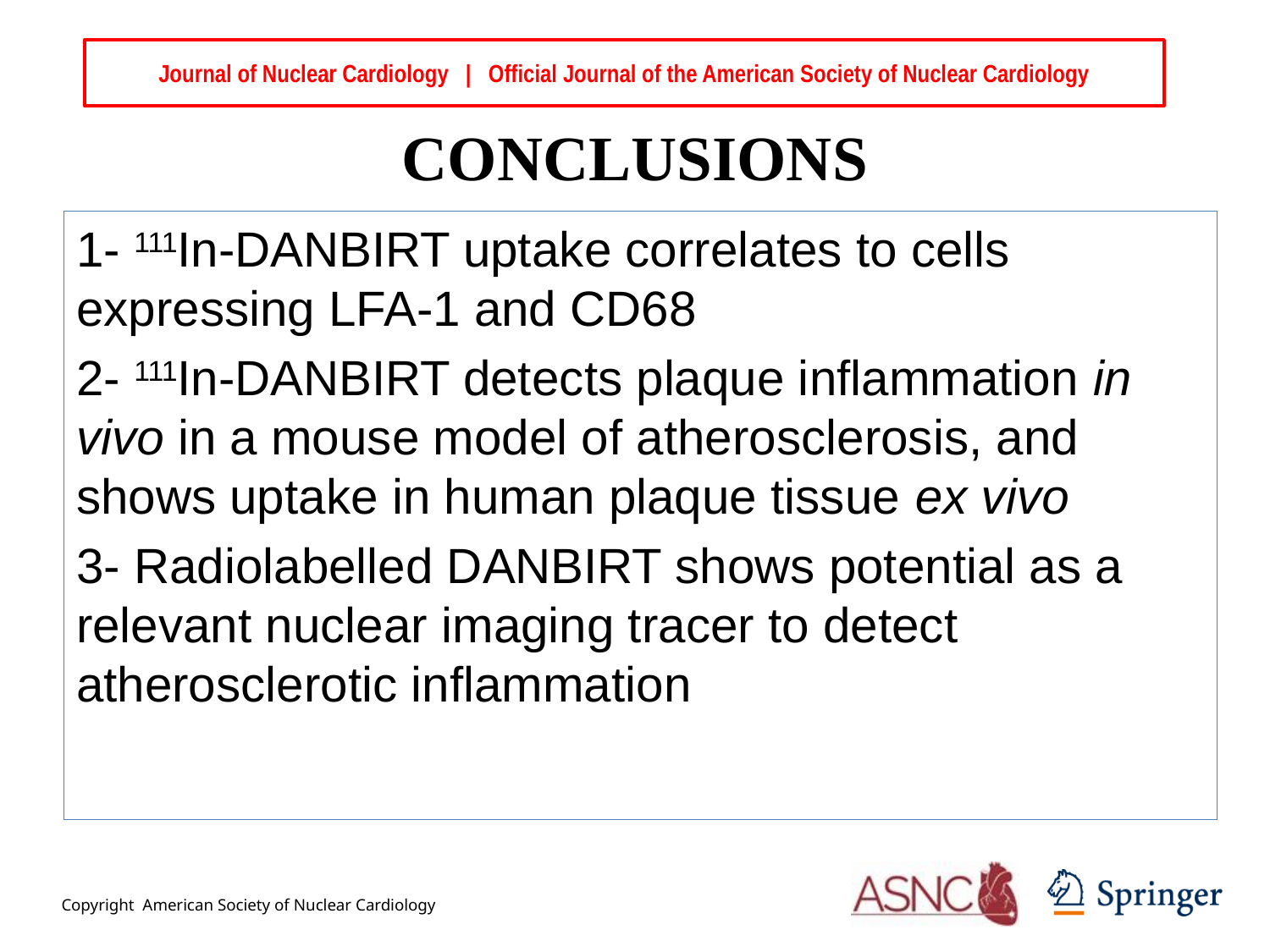

Journal of Nuclear Cardiology | Official Journal of the American Society of Nuclear Cardiology
# CONCLUSIONS
1- 111In-DANBIRT uptake correlates to cells expressing LFA-1 and CD68
2- 111In-DANBIRT detects plaque inflammation in vivo in a mouse model of atherosclerosis, and shows uptake in human plaque tissue ex vivo
3- Radiolabelled DANBIRT shows potential as a relevant nuclear imaging tracer to detect atherosclerotic inflammation
Copyright American Society of Nuclear Cardiology
